# Metabolomic Characterization of Fatty Acids in Patients With Coronary Artery Ectasias

**DOI:** 10.3389/fphys.2021.770223

**Published:** 2021-11-19

**Authors:** Tianlong Liu, Yingying Sun, Hao Li, Haochen Xu, Ning Xiao, Xuliang Wang, Li Song, Congxia Bai, Hongyan Wen, Jing Ge, Yinhui Zhang, Weihua Song, Jingzhou Chen

**Affiliations:** ^1^State Key Laboratory of Cardiovascular Disease, Fuwai Hospital, National Center for Cardiovascular Diseases, Chinese Academy of Medical Sciences and Peking Union Medical College, Beijing, China; ^2^Department of Pharmacy, Affiliated Hospital of Inner Mongolia Medical University, Hohhot, China; ^3^Department of Cardiology, Fuwai Hospital, National Center for Cardiovascular Diseases, Chinese Academy of Medical Sciences and Peking Union Medical College, Beijing, China

**Keywords:** plasma metabolomic profiles, coronary artery ectasia, biomarkers, coronary artery disease, fatty acid metabolites

## Abstract

**Background:** We used a targeted metabolomics approach to identify fatty acid (FA) metabolites that distinguished patients with coronary artery ectasia (CAE) from healthy Controls and patients with coronary artery disease (CAD).

**Materials and methods:** Two hundred fifty-two human subjects were enrolled in our study, such as patients with CAE, patients with CAD, and Controls. All the subjects were diagnosed by coronary angiography. Plasma metabolomic profiles of FAs were determined by an ultra-high-performance liquid chromatography coupled to triple quadrupole mass spectrometric (UPLC-QqQ-MS/MS).

**Results:** Ninety-nine plasma metabolites were profiled in the discovery sets (*n* = 72), such as 35 metabolites of arachidonic acid (AA), eicosapentaenoic acid (EPA), and docosahexaenoic acid (DHA), 10 FAs, and 54 phospholipids. Among these metabolites, 36 metabolites of AA, EPA, and DHA showed the largest difference between CAE and Controls or CAD. 12-hydroxyeicosatetraenoic acid (12-HETE), 17(*S*)-hydroxydocosahexaenoic acid (17-HDoHE), EPA, AA, and 5-HETE were defined as a biomarker panel in peripheral blood to distinguish CAE from CAD and Controls in a discovery set (*n* = 72) and a validation set (*n* = 180). This biomarker panel had a better diagnostic performance than metabolite alone in differentiating CAE from Controls and CAD. The areas under the ROC curve of the biomarker panel were 0.991 and 0.836 for CAE versus Controls and 1.00 and 0.904 for CAE versus CAD in the discovery and validation sets, respectively.

**Conclusions:** Our findings revealed that the metabolic profiles of FAs in the plasma from patients with CAE can be distinguished from those of Controls and CAD. Differences in FAs metabolites may help to interpret pathological mechanisms of CAE.

## Background

Coronary artery ectasia (CAE) is characterized as a diffuse, saccular, irregular, or fusiform dilation of the coronary arteries exceeding 1.5-fold the diameter of the normal adjacent vessel (Eitan and Roguin, 2016). The incidence of CAE was estimated to be 0.5–5%, with male predominance (Yetkin and Waltenberger, 2007). Previous studies reported that coronary luminal enlargement was considered an important reason for angiographic stigmata of impaired blood flow, such as sluggish circulation, swirling, strikingly slow, and scattered clearance of contrast material (Kruger et al., 1999). Indeed, some authors illustrated evidence of stable angina, positive treadmill test, increased levels of biochemical markers, or even myocardial infarction in isolated CAE without obstructive coronary artery disease (CAD), 38.7% of patients with isolated CAE were reported as having a history of myocardial infarction in the corresponding myocardial territory (Demopoulos et al., 1997; Sayin et al., 2001; Manginas and Cokkinos, 2006). More recently, in 2017, Takahito Doi’s study found that the patients with isolated CAE had a significantly higher risk for cardiovascular events than patients without CAE (Doi et al., 2017). Besides, massive enlargement of the coronary artery cannot only result in compression of adjacent structures, vasospasm, thrombosis, and dissection, but also aneurysm rupture, albeit rare can cause acute cardiac tamponade (Kawsara et al., 2018). Therefore, it is clear that CAE, especially the giant ones, is not a benign disease.

Management of patients with CAE remained significant challenge for several reasons: the pathogenesis of CAE is largely unknown. Previous studies showed that 70–80% of CAE were attributed to atherosclerosis and genetic factors, whereas only 10–20% of CEA were associated with inflammatory or connective tissue diseases. Although degradation of the extracellular matrix, nitric oxide dysfunction, and abnormal matrix metalloproteinase activity was recognized as causes of CAE, detailed pathological mechanism still remains unclear (Bergman et al., 2007; Eitan and Roguin, 2016). Also, most CAEs are clinically silent and are only detected incidentally during coronary angiography or CT, while clinically symptomatic isolated CAE-induced myocardial infarction needs to perform percutaneous coronary interventions (Yip et al., 2002). Besides, no specific biomarker for CAE has yet been found, which also represents a huge barrier for further understanding of the mechanisms of CAE (Li et al., 2009).

Fatty acids (FAs) and metabolites played a critical role in the pathogenesis of CAE. Usama Boles’s study found that serum FA metabolites were different in isolated CAE compared to atherosclerosis in mixed CAE, which suggested potentially specific pathophysiology in isolated CAE (Boles et al., 2017). Besides, abnormal FA metabolisms in plasma suggested alterations in lipid signaling in patients with CAE, especially in arachidonic acid (AA) and its metabolites (Zhang et al., 2014). Lipid signaling in the AA cascade is important for regulating some important biological processes, such as inflammation, blood flow, and plaque formation (Buczynski et al., 2009; Watkins and Hotamisligil, 2012). In addition to lipid signaling, FA metabolites, as a component of the phospholipid membrane, play an important role in cell signal transduction (Zeldin, 2001). Furthermore, a previous study showed that polyunsaturated FAs from P-450 metabolites of FAs could also regulate cardiac function and vascular tone (Roman, 2002).

Based on the important roles of FA metabolites in CAE formation, a targeted metabolomics approach was used to discover and subsequently validate the metabolic signatures of these FAs in plasma and to assess the performance of a biomarker signature to distinguish patients with CAE from healthy Controls and patients with CAD, which provided a theoretical basis for further interpreting pathological mechanism of CAE.

## Materials and Methods

Detailed methods are available in the online-only data Supplementary Material.

## Results

### Study Design and Baseline Patient Characteristics

**FIGURE 1:**
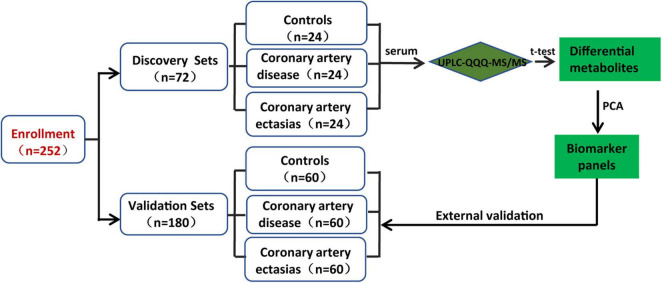
Design of the study.

Two hundred and fifty-two participants were enrolled in the discovery and validation sets. The discovery sets included 72 participants, i.e., 24 participants with CAE and 48 sex-, age- and body mass index (BMI)-matched Controls and participants with CAD. For patients with CAD, the percentages of single-, double-, and multiple-vessel CAD were 29.16, 8.33, and 20.83%, respectively. The patients with CAE were diagnosed by coronary angiography, and CAE was defined as a localized dilatation in the diameter of a coronary artery segment that exceeded the luminal area of the adjacent normal coronary vessels by 1.5-fold. Controls were determined to not have CAE by coronary angiography. The baseline characteristics of the discovery sets are shown in Table 1. Validation sets included 180 participants, and the baseline characteristics of the participants are shown in Supplementary Table 2. The workflow of the study is presented in Figure 1.

**TABLE 1:** Baseline characteristics of patients in the discovery sets.

	Controls	CAD	CAE	*p* Value for trend
		
*n*	24	24	24	
Age, yrs	54.42 ± 10.15	53.96 ± 11.17	54.58 ± 9.86	0.484
Men, %	66.7	66.7	66.7	
BMI, kg/m^2^	26.25 ± 2.83	26.08 ± 3.11	26.67 ± 2.87	0.272
SBP, mm Hg	124.6 ± 14.86	128.2 ± 17.56	131.6 ± 20.46	0.350
DBP, mm Hg	79.29 ± 9.26	77.656 ± 12.36	82.38 ± 13.18	0.264
TC, mmol/L	4.19 ± 0.94	4.00 ± 1.09	4.15 ± 1.34	0.260
TG, mmol/L	1.41 ± 0.55	1.67 ± 1.08	1.53 ± 0.63	0.0043
HDL-C, mmol/L	1.12 ± 0.48	1.18 ± 0.87	0.99 ± 0.30	<0.0001
LDL-C, mmol/L	2.67 ± 0.90	2.52 ± 0.84	2.77 ± 1.17	0.248
Glucose, mmol/L	5.62 ± 1.55	5.83 ± 1.35	5.38 ± 1.08	0.240
Cigarette smoking, %				0.153
Never	39.13	62.5	37.5	
Current	60.89	37.5	62.5	
Alcohol intake, %				0.252
Never	52.17	79.17	60.54	
Current	47.83	20.83	39.46	
Hypertension history, %	66.67	62.50	70.83	0.829
DM history, %	12.50	12.50	8.33	0.869

*Age, body mass index (BMI), Systolic (SBP) and diastolic (DBP) blood pressure, glucose, and TC values are given as means (±SD); TG values are medians (range), and the number of individuals (n) with percentage (n/N) are indicated. DM indicates Diabetes Mellitus. Body mass index is calculated as individual’s body weight divided by the square of individual’s height.*

### Metabolic Profiles of Fatty Acids in Plasma From Different Subjects

**FIGURE 2:**
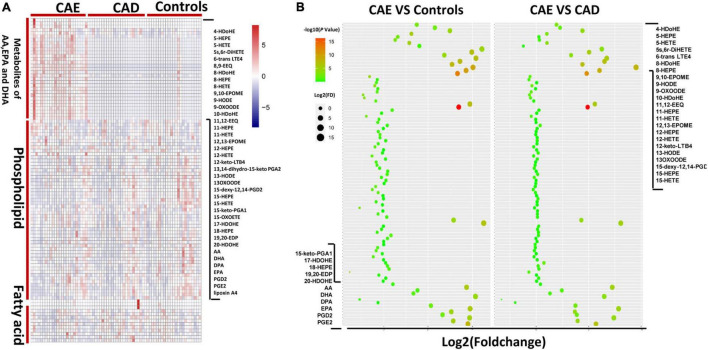
Metabolic profiles of lipids in plasma from different subjects. **(A)** Comparison of 99 plasma metabolites concentrations in 72 discovery sets. **(B)** Fold change of 99 plasma metabolites in 72 discovery sets.

To evaluate differences in FA metabolisms between subjects with CAE versus Controls or patients with CAD, we first performed metabolic profiling of FAs in plasma from different subjects. In total, 99 plasma metabolites were profiled in the discovery sets (*n* = 72), such as 35 metabolites of AA, eicosapentaenoic acid (EPA), and docosahexaenoic acid (DHA), 10 FAs, and 54 phospholipids (Figure 2A). For 35 metabolites of AA, EPA, and DHA, a total of 28 and 29 metabolites were significantly different between CAE versus Controls and CAE versus CAD, respectively (*P* < 0.05), although no significantly different metabolites were found between CAD versus Controls (Figure 2B and Supplementary Table 3). A total of 10, 5, and 3 phospholipids were significantly different between CAE versus Controls, CAE versus CAD, and CAD versus Controls, respectively (*P* < 0.05; Supplementary Table 4). A total of 3 and 4 FAs were significantly different between CAE versus Controls and CAE versus CAD, respectively (*P* < 0.05), however, no significantly different metabolites were found between CAD versus Controls (Supplementary Table 5). Therefore, 35 metabolites of AA, EPA, and DHA were emerged as the metabolites with the most significant differences between CAE and Controls or CAE and CAD compared with phospholipids and FAs.

### Defining of a Potential Metabolic Biomarker for Coronary Artery Ectasia

**FIGURE 3:**
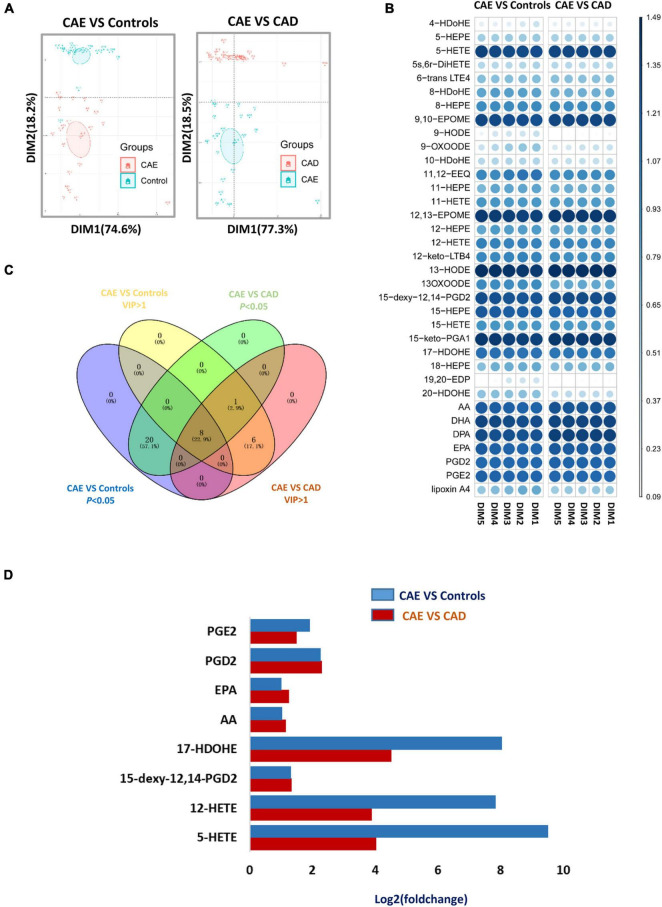
Defining of a potential metabolic biomarker for CAE. **(A)** Score plot of the first (PC1) and second (PC2) PCs (horizontal and vertical axes, respectively) from principal component analysis of arachidonic acid metabolites in discovery sets. **(B)** VIP value of metabolites from PCA analysis in discovery sets. **(C)** Venn diagram shows an overlap between metabolites with VIP value > 1 and *P*-value < 0.05. **(D)** Fold change of eight serum metabolites from C in discovery sets. CAE, coronary artery ectasia; PCA, principal component analysis.

Principal component analysis (PCA) score plots revealed that subjects with CAE were separated from Controls and subjects with CAD (Figure 3A). Fourteen and 15 metabolites with VIP (variable importance in projection) value > 1 on two principal components were found as important metabolites for distinguishing CAE from Controls and CAD, respectively (Figure 3B and Supplementary Table 6). Eight metabolites were screened as biomarker candidates via overlapping VIP value and *P*-value (Figure 3C). These metabolites were significantly increased in serum of patients with CAE compared with patients with CAD and Controls (Figure 3D). To further validate the eight biomarker candidates screened from the discovery sets, an independent validation set (*n* = 180) was used, and these metabolites were detected by target metabolomics. The following criteria were satisfied to screen useful biomarkers: (1) *P* < 0.05 for CAE versus Controls and CAE versus CAD, respectively; and (2) having the same change trend as the discovery sets. Finally, five metabolites were screened: 12-hydroxyeicosatetraenoic acid (12-HETE), 17(*S*)-hydroxydocosahexaenoic acid (17-HDoHE), EPA, AA, and 5-HETE (Supplementary Table 7).

### Validation of the Diagnostic Performance of the Biomarker Panel for Coronary Artery Ectasia

**FIGURE 4:**
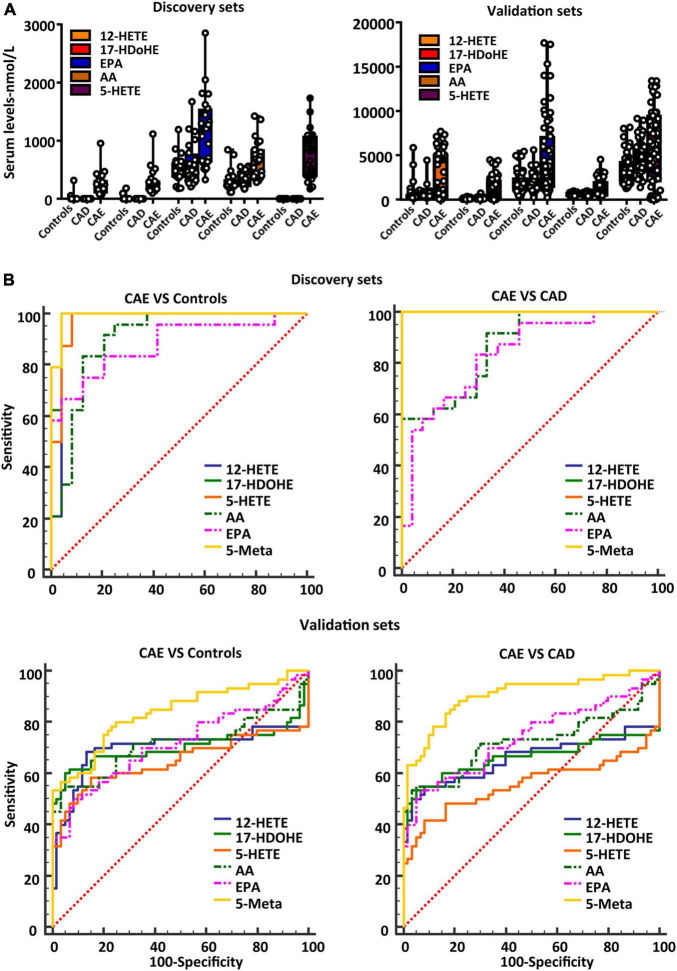
Validating the diagnostic performance of the biomarker panel for CAE. **(A)** Serum concentrations of a defined biomarker panel in the discovery and validation sets. **(B)** ROC curves of biomarker panel and signal metabolites in discovery sets and validation sets. CAE, coronary artery ectasia.

To further validate the diagnostic performance of the biomarker panel for CAE, a validation set (*n* = 180) was used. The serum concentrations of the biomarker panel are shown in Figure 4A. Biomarker panels were used to validate the diagnostic performance via a logistical regression model. The results showed that the biomarker panel had better diagnostic accuracy than signal metabolites for CAE. The areas under the ROC curve of the biomarker panel were 0.991 and 0.836 for CAE versus Controls, 1.00 and 0.904 for CAE versus CAD in the discovery and validation sets, respectively (Figure 4B and Table 2).

**TABLE 2:** Results of measurement of the serum metabolite panel in the diagnosis of CAE.

	Discovery sets (*n* = 72)			Validation sets (*n* = 180)		
	AUC (95% CI)	Sensitivity (%)	Specificity (%)	AUC (95% CI)	Sensitivity (%)	Specificity (%)
**CAE versus Controls**						
12-HETE	0.967 (0.870–0.997)	100	98.5	0.698 (0.608–0.778)	68.3	86.7
17-HDoHE	0.984 (0.898–1.000)	100	95.8	0.703 (0.613–0.783)	60.0	96.7
AA	0.901 (0.780–0.968)	95.8	75.0	0.714 (0.624–0.793)	55.0	95.0
EPA	0.88 (0.754–0.956)	83.3	79.2	0.715 (0.625–0.793)	50.0	91.7
5-HETE	0.974 (0.881–0.999)	100	91.7	0.648 (0.555–0.733)	58.33	85.00
5-Meta	0.991 (0.910–1.000)	100	95.83	0.836 (0.757–0.897)	80.00	75.00
**CAE versus CAD**						
12-HETE	1 (0.926–1.000)	100	100	0.658 (0.566–0.742)	55.0	91.7
17-HDoHE	1 (0.926–1.000)	100	100	0.659 (0.566–0.743)	50.0	98.3
AA	0.868 (0.739–0.948)	58.3	100	0.721 (0.632–0.799)	55.0	95.0
EPA	0.837 (0.702–0.928)	83.3	70.8	0.739 (0.651–0.815)	55.3	93.3
5-HETE	1.00 (0.926–1.00)	100	100	0.558 (0.465–0.649)	41.67	91.67
5-Meta	1 (0.926–1.000)	100	100	0.904 (0.836–0.950)	86.67	80.00

*CAE, coronary artery ectasia; CI, confidence interval; 5-Meta, serum metabolite panel.*

## Discussion

In our study, a target metabolomics approach was employed to analyze the metabolic profile characteristics of FAs in patients with CAE. PCA analysis showed good discrimination of CAE from Controls or CAD in the discovery sets by using 36 metabolites of AA, EPA, and DHA. Overall, a biomarker panel, such as 12-HETE, 17-HDoHE, EPA, AA, and 5-HETE, was identified for distinguishing CAE from Controls and CAD in the discovery. The diagnostic activity of the biomarker panel for distinguishing CAE from CAD and Controls was verified in the validation sets.

Although approximately half of CAE occurred due to atherosclerosis, a minority of cases was observed in the absence of a significant atherosclerotic lesion (Bilik et al., 2015). Patients with isolated CAE exhibited distinct clinical characteristics, such as more frequent involvement of the right coronary artery and a lower frequency of stent implantation (ElGuindy and ElGuindy, 2017). Moreover, patients with CAE coexisting with CAD had no additional risk of cardiovascular events compared to those with CAD only, however, even patients with isolated CAE had a significantly increased risk for cardiovascular events due to slow coronary blood flow, microemboli or thrombosis (Yetkin and Waltenberger, 2007). Besides, Usama Boles’s study found that serum lipid profiles were different in isolated CAE compared to atherosclerosis in mixed CAE, which suggested potentially specific pathophysiology in isolated CAE (Boles et al., 2017). Therefore, it is not fully justified to conclude that CAE is a subtype of coronary atherosclerosis. However, the clinical presentation of CAE and CAD was similar, such as ischemic cardiomyopathy, unstable angina, and myocardial infarction. Therefore, healthy subjects and patients with CAD as Control were enrolled in our study, which was helpful to improve the accuracy and specificity of our results.

Unsaturated FAs and metabolites as potent endogenous mediators were involved in regulating various biological processes, such as inflammation, pain, and blood coagulation (Schuchardt et al., 2013). AA (C20:4) as omega-6 FAs, EPA (20:5 n-3), and DHA (22:6 n-3) as omega-3 FAs were essential polyunsaturated FA and rely largely on the dietary intake for low conversion rate in adult (Brenna et al., 2009). AA, DHA, and EPA were catalyzed by lipoxygenases to form regio- and stereo-selective hydroperoxides and then were reduced to HETEs, HDoHE, and HEPEs (hydroxy-6E,8Z,11Z,14Z,17Z-eicosapentaenoic acid), respectively (Brash, 1999). Previous studies showed that those oxylipins not only serve as precursors for leukotrienes and hepoxylins that played a critical role in regulating inflammation reaction and coagulation process, but also themselves and downstream products, such as oxo-ETE (oxo-6E,8Z,11Z,14Z-eicosatetraenoic acid) from HETEs could regulate various biological processes via G protein-coupled receptors pathway (Powell and Rokach, 2013). In our study, a significant increase in serum 12-HETE, 17-HDoHE, EPA, AA, and 5-HETE level was found in patients with CAE compared with patients with CAD and Controls, moreover, those metabolites as biomarker panel could be used to distinguish CAE from Controls and CAD in the discovery and validation sets.

Local alteration in coronary tone was involved in the pathological process of CAE (Yetkin and Waltenberger, 2007). Previous studies showed that vessel endothelial cells could synthesize and release CYP450-derived FA metabolites as endothelium-derived hyperpolarizing factors led to the hyperpolarization and relaxation of smooth muscle cells (SMC) by activating Ca^2+^-dependent K^+^ channels and the Na-K-ATPase pathway (Malmsjo et al., 1999). Moreover, CYP450 inducers can regulate SMC hyperpolarization to relax the coronary artery by increasing the synthesis of FA metabolites (Fleming, 2000). Our targeted metabolic profile showed that the FA metabolites level in plasma was significantly increased in patients with CAE compared to those in Controls and patients with CAD. We speculated that those increased metabolites led to a local alteration in coronary tone by an endothelium-independent pathway (Nishikawa et al., 1999).

Fatty acid metabolites are closely associated with nitric oxide (NO) release, while NO overstimulation and medial thinning were important pathological mechanisms leading to CAE (Sorrell et al., 1996). FA metabolites as endothelium-derived hyperpolarizing factors could mediate endothelium-dependent relaxations via promoting endothelial nitric oxide synthase (eNOS) expression and NO release (Zuccolo et al., 2016). Indeed, EPA via upregulation of uncoupling protein 2 activates AMPKα1 resulting in increased endothelial nitric oxide synthase (NOS) phosphorylation and promoted NO release in aortic endothelial cells (Wu et al., 2012). Meanwhile, W. Raphael’s study found that mRNA expression level of NOS_2_ in leukocytes had a close association with plasma oxylipid concentrations, especially for 9-HODE and 13-HODE, while 13-HODE as a substrate was oxidized to the relatively stable 13-Oxo-ODE (Ramsden et al., 2012; Raphael et al., 2014). Our results showed that higher concentrations of FA metabolites in the peripheral blood of patients with CAE than in that of Controls and CAD might contribute to CAE formation via the NO pathway.

A previous study showed that 10–20% of CEA have been described in association with inflammatory or connective tissue diseases, while FAs and their derivatives link nutrient metabolism to inflammation reaction (Sonnweber et al., 2018). During the early phase of inflammation, AA is predominantly metabolized via 5-lipoxygenase (5-LOX), which produces pro-inflammatory leukotriene, such as leukotriene B4 (LTB4), whereas in the late phase prostaglandin E2, enhance 15-LOX expression, followed by a switch from LTB4 synthesis to 5-LOX and 15-LOX-mediated lipoxin A4 production, which contribute to local inflammation (Ho et al., 2010). A previous study showed that 5-LOX and 15-LOX were crucial enzymes that helped in the conversion of AA to 5-HETE (Sinha et al., 2019). In our study, AA, 17-HDoHE, and 5-HETE had a significant increase in the peripheral blood of patients with CAE compared to patients with CAD and Controls, these metabolites might contribute to CAE formation via an inflammatory pathway.

Another pathological mechanism of CAE is related to the metalloproteinase system (Manginas and Cokkinos, 2006). On the one hand, gene polymorphisms of matrix metalloproteinase (MMP)-3 were significantly different in patients with CAE compared to patients with coronary lesions. On the other hand, cardiac-specific over-expression of MMP-2 could induce CAE in mice (Dahi et al., 2011). 9-hydroxyoctadecadienoic acid (9-HODE) was reported to promoting the expression of metallopeptidase domain 17 to induce SMC apoptosis, extracellular matrix degradation, and necrotic core growth (Garbin et al., 2013; Vendrov et al., 2017). Recent studies have documented that 20-HETE as a CYP450-derived AA metabolite was correlated with increased elastin degradation by activating MMP-12 in Ang II-independent pathways (Soler et al., 2018). In addition to 20-HETE, 15-HETE could also induce MMP expression in vessel endothelial cells *in vivo* and *in vitro* (Prato et al., 2010; Liu et al., 2018). Therefore, FA metabolites may induce CAE formation by activating metalloproteinase.

In summary, the present study shows that the plasma FA profiles of patients with CAE could be seen as biomarkers to distinguish CAE from Controls and patients with CAD. Moreover, the diagnostic accuracy of the metabolic biomarkers was verified in the validation sets. Characterizing the metabolic profile of FAs in the peripheral blood from patients with CAE may help to comprehend the underlying biological mechanisms of the disease.

## Data Availability Statement

The original contributions presented in the study are included in the article/Supplementary Material, further inquiries can be directed to the corresponding authors.

## Ethics Statement

The studies involving human participants were reviewed and approved by the protocol was approved by the Institutional Review Board of Fuwai Hospital (Approval No: 2018-1066). All subjects gave written informed consent in accordance with the Declaration of Helsinki. The protocol was approved by the Fuwai Hospital. The patients/participants provided their written informed consent to participate in this study. The animal study was reviewed and approved by the protocol was approved by the Institutional Review Board of Fuwai Hospital (Approval No: 2018-1066). All subjects gave written informed consent in accordance with the Declaration of Helsinki. The protocol was approved by the Fuwai Hospital.

## Author Contributions

TL, YS, HL, and HX worked on sample preparation, data analysis, and drafted the manuscript. NX, XW, LS, CB, HW, and JG conducted sample management. YZ, WS, and JC worked on experimental design, sample management, data analysis, and manuscript preparation. All authors contributed to the article and approved the submitted version.

## Conflict of Interest

The authors declare that the research was conducted in the absence of any commercial or financial relationships that could be construed as a potential conflict of interest.

## Publisher’s Note

All claims expressed in this article are solely those of the authors and do not necessarily represent those of their affiliated organizations, or those of the publisher, the editors and the reviewers. Any product that may be evaluated in this article, or claim that may be made by its manufacturer, is not guaranteed or endorsed by the publisher.
