# Supplementary material for: Metabolomic Characterization of Fatty Acids in Patients With Coronary Artery Ectasias
Source: Front Physiol. 2021 Nov 19;12:770223. doi: 10.3389/fphys.2021.770223 (PMC8640203; doi:10.3389/fphys.2021.770223)
Supplement: Supplementary file 2 [file Data_Sheet_1.docx]

**Supplement materials**

**Metabolomic Characterization of Fatty Acids in Patients with Coronary Artery Ectasias**

Tianlong Liu^1,2*^, Yingying Sun^1*^, Hao Li^1^, Haochen Xu^1^, Ning Xiao^1^, Xuliang Wang^1^, Li Song ^1^, Congxia Bai^1^, Hongyan Wen^1^, Jing Ge^1^, Yinhui Zhang^1^, Weihua Song^3^ and Jingzhou Chen^1^

**Author affiliations:**

* contributed equally

^1^State Key Laboratory of Cardiovascular Disease, Fuwai Hospital, National Center for Cardiovascular Diseases, Chinese Academy of Medical Sciences and Peking Union Medical College, Beijing 100037, China.

^2^Department of Pharmacy, Affiliated Hospital of Inner Mongolia Medical University, 010059 Hohhot, PR China.

^3^ Department of Cardiology, Chinese Academy of Medical Sciences and Peking Union Medical College, National Center for Cardiovascular Diseases, Fuwai Hospital, Beijing 100037, China.

**Correspondence to:**

Weihua Song, MD

Department of Cardiology, Chinese Academy of Medical Sciences and Peking Union Medical College, National Center for Cardiovascular Diseases, Fuwai Hospital, 167 Beilishilu, Xicheng District, Beijing 100037, China.

E-mail: songweihua926@163.com

Phone: Tel: +8610 88396590.

Jingzhou Chen, PhD

State Key Laboratory of Cardiovascular Disease, Fuwai Hospital, National Centre for Cardiovascular Diseases, Chinese Academy of Medical Sciences and Peking Union Medical College, 167 Beilishilu, Xicheng District, Beijing 100037, China.

E-mail: chendragon1976@aliyun.com

Phone: Tel: +8610 88396197; fax: +8610 68331730;

**Running Title:** Metabolic Characterization of arachidonic acid metabolites and Coronary Artery Ectasias

**MATERIALS AND METHODS**

This study was performed in accordance with the Declaration of Helsinki and was approved by the Institutional Review Board of Fuwai Hospital (Approval NO: 2016-732). All participants gave their written informed consent.

**Study participants**

Two hundred and fifty-two patients in this study were symptomatic and underwent coronary angiography for suspected ischemic heart disease. CAE was diagnosed and confirmed by coronary angiography, as well as CAD and controls. The angiogram of each patient was screened by 2 experienced interventional cardiologists. Shortly after the coronary angiography (within a few days), the patients were enrolled in this study. Detailed inclusion criteria were (1) CAE: coronary abnormal dilation of more than 1.5-fold the diameter of adjacent normal segments, with no stenosis in coronary angiography. (2) CAD: stenosis is present of at least 50% in one major native epicardial coronary artery with a diameter of at least 2mm in coronary angiography. (3) Normal: no CAE, CAD or any other abnormal findings in coronary angiography. The exclusion criteria were (1) coronary artery fistula; (2) stent-related coronary artery aneurysms; (3) known autoimmune disease; (4) valvular heart disease; or (5) history of coronary artery bypass graft. Controls and patients with CAD were matched according to the age and sex of the patients with CAE. Blood samples from the patients and controls were stored in K2 EDTA Vacutainer tubes and cooled down in a refrigerator (4 °C) immediately. They were then centrifuged at 3000 × g for 10 min at 4 °C within 2 h. Supernatants (plasma) were separated, transferred into new vials, immediately frozen and stored (80 °C) until sample preparation.

**Metabolomics analysis**

**Reagents and materials**

In total, 110 AA-derived eicosanoids and 5 deuterium-labelled internal standards (ISs) were purchased from Cayman Chemical (Ann Arbor, MI, USA). One hundred and ten eicosanoid standard compounds and their fragment ions, retention time and internal standards were showed in **Supplemental Table S1**. All other chemicals were purchased from Sigma-Aldrich (St. Louis, MO, USA).

**Sample preparation**

The sample extraction method was taken from literature(Blanchard, 1981; Masoodi et al., 2010; Strassburg et al., 2012; Wang et al., 2014). Plasma samples (total volume of 80 μL) and 10 μL of butylhydroxytoluene (BHT)/MeOH solution (W:V, 4.8 g/100 mL) were subjected to protein precipitation by adding 100 μL of MeOH containing deuterium-labelled ISs, at a final concentration of 50 ng/mL each of prostaglandin E2-d4, 6-keto prostaglandin F1α-d4, 5(S)-HETE-d8, 9(S)-HODE-d4 and 200 ng/mL arachidonic acid-d8. Samples were centrifuged at 12000 rpm for 10 min at 4 °C, and the supernatants were transferred to new tubes, followed by dilution with pure water to 15% methanol concentration (containing 0.005% formic acid) and solid phase extraction (SPE) after pretreatment with MeOH and equilibration with H2O. The extraction was dried and re-dissolved in 100 μL of MeOH, and then the solution was filtered through a 0.22 µm membrane filter.

**Liquid chromatography and mass spectrometry**

Samples were analysed by liquid chromatography (Agilent 1290, San Jose, CA, USA) coupled to electrospray ionization on a triple quadrupole mass spectrometer (Agilent 6470). For the analysis, 3 μL of the extraction was injected. The auto-sampler was cooled at 4 °C. Chromatographic separation was achieved on an Agilent ZORBAX RRHD Eclipse XDB C18 column (2.1×100 mm, 1.8 µm particles) using a flow rate of 0.65 mL/min at 45 °C over a 13-min gradient (0-12 min from 68% A to 20% A, 12-13 min at 5% A), while using the solvents A, water containing 0.005% formic acid, and B, acetonitrile containing 0.005% formic acid. Electrospray ionization was performed in negative ion mode using N_2_ at a pressure of 30 psi for the nebulizer. The dry gas with a flow of 10 L/min and a temperature of 300 °C. The sheath gas temperature was 350 °C with a flow rate of 11 L/min. The capillary was set at 3500 V, and the nozzle voltage was 500 V. Multiple reaction monitoring (MRM) was used for the quantification of screening fragment ions.

**Calibration curves**

Calibration curve linearity was evaluated by assessing the correlation coefficients (R^2^). Standard curves were constructed by least-squares linear regression analysis using the peak area ratio of a given eicosanoid over its reference IS against the nominal concentration of the calibrator.

**Data preprocessing**

Peak determination and peak area integration were performed with Mass Hunter (Agilent, Version B.08.00), while auto-integration was manually inspected and corrected if necessary. The concentration of each analyte was calculated by interpolation of the observed analyte/IS peak-area ratio.

**Statistical analysis**

Data are displayed as the means ± standard deviations (SDs) for continuous variables. Statistical comparisons for percentages were performed using χ^2^ analysis. Principal component analysis (PCA) and Bayesian factor analysis (with normalized factor score plots) were utilized to determine the main sources of inter-individual variation in the plasma metabolome. In addition to the multivariate statistical method, Student’s t-test was also applied to measure the significance of each metabolite. Logistic regression analysis and receiver-operating characteristic (ROC) analysis were used for the diagnosis of different CAD stages. Statistical analyses were performed using SPSS software version 19.0 (IBM Corp., New York). An adjusted P value of <0.05 was considered indicative of statistical significance.

**Supplemental Table S2** Baseline characteristics of validation sets

|  | Controls | CAD | CAE | *P* Value for Trend |
| --- | --- | --- | --- | --- |
| n | 180 | 180 | 180 |  |
| Age, yrs | 57.93±6.96 | 58.15±7.08 | 58.05±6.89 | 0.98 |
| Men, % | 81.67 | 81.67 | 81.67 |  |
| BMI, kg/m2 | 25.44±2.81 | 25.38±3.22 | 26.73±3.82 | 0.51 |
| SBP, mm Hg | 117.1±17.42 | 111.8±13.62 | 129.0±13.95 | <0.01 |
| DBP, mm Hg | 82.35±15.25 | 83.61±18.62 | 80.16±13.68 | <0.01 |
| TC, mmol/L | 4.68±1.30 | 4.07±0.97 | 4.36±1.28 | 0.0127 |
| TG, mmol/L | 2.20±1.90 | 1.73±0.88 | 1.95±0.90 | 0.330 |
| HDL-C, mmol/L | 1.15±0.32 | 1.09±0.30 | 0.98±0.22 | 0.0058 |
| LDL-C, mmol/L | 2.73±0.95 | 2.33±0.80 | 2.54±0.97 | 0.289 |
| Glucose, mmol/L | 5.37±1.03 | 5.81±2.04 | 5.45±1.39 | 0.9686 |
| Cigarette smoking, % |  |  |  | 0.004 |
| Never | 54.2 | 51.7 | 29.3 |  |
| Current | 45.8 | 48.3 | 70.7 |  |
| Alcohol intake, % |  |  |  | 0.233 |
| Never | 59.32 | 76.67 | 50.94 |  |
| Current | 40.68 | 23.33 | 49.06 |  |
| Hypertension history, % | 43.33 | 66.67 | 81.03 | 0.000 |
| DM history, % | 11.67 | 25.00 | 17.24 | 0.334 |

Age, body mass index (BMI), Systolic (SBP) and diastolic (DBP) blood pressure, glucose, and TC values are given as means (±SD); TG values are medians (range), and the number of individuals (n) with percentage (n/N) are indicated. DM indicates Diabetes Mellitus. Body mass index is calculated as individual’s body weight divided by the square of individual’s height.

**Supplemental Table S3** Statistical analysis of metabolites of arachidonic acid(AA), eicosapentaenoic acid(EPA) and docosahexaenoic acid(DHA) in discovery sets

| Metabolites | CAE versus Controls | | | Metabolites | CAE versus CAD | | |
| --- | --- | --- | --- | --- | --- | --- | --- |
|  | FC | log2(FC) | *P* Value |  | FC | log2(FC) | *P* Value |
| 19,20-EDP | 10.457 | 3.3864 | 7.79E-19 | 19,20-EDP | 29.759 | 4.8952 | 5.51E-26 |
| 4-HDoHE | 10.047 | 3.3287 | 1.49E-13 | 4-HDoHE | 27.941 | 4.8043 | 4.34E-17 |
| 5-HEPE | 13.12 | 3.7137 | 6.23E-11 | 5-HEPE | 65.27 | 6.0283 | 7.35E-13 |
| 5-HETE | 16.303 | 4.0271 | 2.79E-10 | 5-HETE | 730.8 | 9.5133 | 4.19E-12 |
| 10-HDoHE | 13.915 | 3.7986 | 5.83E-09 | 5s,6r-DiHETE | 21.111 | 4.3999 | 1.76E-10 |
| 5s,6r-DiHETE | 8.4663 | 3.0817 | 2.30E-08 | 6-trans LTE4 | 91.128 | 6.5098 | 2.27E-10 |
| 20-HDOHE | 14.821 | 3.8896 | 4.10E-08 | 10-HDoHE | 51.637 | 5.6903 | 3.76E-10 |
| 6-trans LTE4 | 10.842 | 3.4386 | 4.20E-08 | 20-HDOHE | 47.9 | 5.5819 | 3.99E-09 |
| 8-HDoHE | 17.887 | 4.1608 | 6.95E-08 | 8-HDoHE | 105.36 | 6.7191 | 9.32E-09 |
| 9-HODE | 15.709 | 3.9735 | 8.01E-08 | 9-HODE | 32.845 | 5.0376 | 1.99E-08 |
| 17-HDOHE | 22.821 | 4.5123 | 2.43E-07 | 17-HDOHE | 265.9 | 8.0547 | 6.87E-08 |
| 12-HETE | 14.917 | 3.8988 | 1.50E-06 | 15-HETE | 151.95 | 7.2474 | 1.15E-07 |
| 15-HETE | 13.359 | 3.7398 | 1.58E-06 | 12-HETE | 230.22 | 7.8468 | 1.35E-07 |
| EPA | 2.3768 | 1.249 | 2.69E-06 | 11-HEPE | 15.389 | 3.9438 | 7.16E-07 |
| 11-HEPE | 8.7952 | 3.1367 | 4.68E-06 | 11-HETE | 168.47 | 7.3963 | 1.67E-06 |
| AA | 2.2251 | 1.1539 | 5.78E-06 | lipoxin A4 | 21.124 | 4.4008 | 1.94E-06 |
| 11-HETE | 14.67 | 3.8748 | 1.13E-05 | 12-HEPE | 12.827 | 3.6811 | 2.89E-06 |
| 12-HEPE | 8.288 | 3.051 | 1.18E-05 | 18-HEPE | 19.675 | 4.2983 | 3.54E-06 |
| 9-OXOODE | 21.567 | 4.4308 | 2.36E-05 | AA | 2.0356 | 1.0254 | 9.68E-06 |
| 18-HEPE | 8.9049 | 3.1546 | 2.83E-05 | 9-OXOODE | 75.942 | 6.2468 | 1.09E-05 |
| lipoxin A4 | 7.9235 | 2.9861 | 2.85E-05 | 13OXOODE | 89.026 | 6.4762 | 1.72E-05 |
| 13OXOODE | 17.644 | 4.1411 | 4.81E-05 | 8-HEPE | 9.9712 | 3.3178 | 1.85E-05 |
| 8-HEPE | 6.6467 | 2.7326 | 7.93E-05 | EPA | 2.0122 | 1.0088 | 0.000108 |
| PGE2 | 2.8104 | 1.4908 | 0.002463 | PGE2 | 3.7995 | 1.9258 | 0.000162 |
| 11,12-EEQ | 6.2933 | 2.6538 | 0.006492 | 12-keto-LTB4 | 13.308 | 3.7343 | 0.000622 |
| PGD2 | 4.9467 | 2.3065 | 0.006575 | 11,12-EEQ | 14.555 | 3.8635 | 0.001719 |
| 12-keto-LTB4 | 4.3761 | 2.1297 | 0.012373 | PGD2 | 4.8273 | 2.2712 | 0.00659 |
| 15-dexy-12,14-PGD2 | 2.5302 | 1.3392 | 0.044375 | 15-HEPE | 5.411 | 2.4359 | 0.021881 |
|  |  |  |  | 15-dexy-12,14-PGD2 | 2.48 | 1.3104 | 0.044805 |

The criteria of differential metabolomics based on fold change >2.0 and *p* value <0.05;

**Supplemental Table S4** Statistical analysis of phospholipid in discovery sets

| **Metabolites** | **CAE versus CAD** | | **CAE versus Controls** | | **CAD versus Controls** | |
| --- | --- | --- | --- | --- | --- | --- |
|  | **Foldchange** | **P value** | **Foldchange** | **P value** | **Foldchange** | **P value** |
| 16:1/18:1-PE | 1.15789568 | 0.45211 | 0.81416387 | 0.26336 | 0.70314095 | 0.069339 |
| 18:1/16:1-PE | 0.99791649 | 0.989492 | 1.13510152 | 0.381998 | 1.13747145 | 0.416149 |
| 18:0/16:1-PE | 1.07943848 | 0.513936 | 0.83730559 | 0.15314 | 0.77568625 | 0.067671 |
| 20:4/16:0-PE | 0.95697135 | 0.801443 | 0.83218783 | 0.305963 | 0.86960579 | 0.473832 |
| 16:0/20:4-PE | 0.86324358 | 0.437351 | 0.72001341 | 0.057198 | 0.83407908 | 0.274777 |
| 18:1/18:2-PE | 0.84407366 | 0.278067 | 0.69269188 | 0.027031* | 0.82065336 | 0.195364 |
| 18:2/18:1-PE | 0.99618014 | 0.979395 | 0.84290068 | 0.319561 | 0.84613279 | 0.380449 |
| 18:2/18:0-PE | 1.06954105 | 0.628686 | 0.89027074 | 0.459335 | 0.83238576 | 0.293981 |
| 18:1/18:1-PE | 0.96355002 | 0.829218 | 0.78767039 | 0.18545 | 0.81746705 | 0.260441 |
| 18:1/18:0-PE | 0.92659494 | 0.637445 | 0.81021275 | 0.15267 | 0.87439798 | 0.417216 |
| 18:0/18:1-PE | 1.05658041 | 0.805795 | 0.78777718 | 0.298249 | 0.74559132 | 0.15648 |
| 18:2/20:4-PE | 0.72040352 | 0.043738* | 0.57945605 | 0.004959* | 0.80434927 | 0.210338 |
| 20:3/18:2-PE | 0.68855589 | 0.029682* | 0.54522297 | 0.002485** | 0.79183546 | 0.195817 |
| 18:1/20:4-PE | 0.84667855 | 0.23879 | 0.76068646 | 0.032155* | 0.89843596 | 0.429629 |
| 18:0/20:4-PE | 0.81972058 | 0.18359 | 0.69909034 | 0.016378* | 0.8528398 | 0.245515 |
| 20:3/20:3-PE | 0.64147291 | 0.007305* | 0.52471409 | 0.000548*** | 0.81798325 | 0.247229 |
| 16:1/16:1-PC | 1.15179871 | 0.196495 | 1.25396566 | 0.062434 | 1.08870208 | 0.396889 |
| 16:0/16:1-PC | 1.17765777 | 0.279466 | 1.06561861 | 0.63661 | 0.90486272 | 0.527602 |
| 16:1/16:0-PC | 0.97688069 | 0.806607 | 1.12392442 | 0.249592 | 1.15052373 | 0.162529 |
| 16:0/16:0-PC | 1.02083019 | 0.814878 | 0.98288513 | 0.85165 | 0.96282921 | 0.671023 |
| 16:1/18:2-PC | 1.17729427 | 0.226751 | 0.99034901 | 0.939368 | 0.8412077 | 0.255017 |
| 16:0/18:3-PC | 1.66118439 | 0.00149* | 1.05984192 | 0.715084 | 0.63800378 | 0.011267* |
| 16:0/18:2-PC | 1.1349991 | 0.197901 | 1.12326185 | 0.219437 | 0.9896588 | 0.896228 |
| 16:0/18:1-PC | 1.19157633 | 0.136434 | 1.18183325 | 0.125897 | 0.99182337 | 0.932561 |
| 18:1/16:0-PC | 1.09766922 | 0.25668 | 1.11331661 | 0.166551 | 1.01425511 | 0.866282 |
| 18:0/16:0-PC | 1.08194821 | 0.333436 | 1.10096781 | 0.213231 | 1.01757903 | 0.83518 |
| 16:0/18:0-PC | 1.01645659 | 0.910949 | 0.85798396 | 0.38697 | 0.84409307 | 0.314186 |
| 16:0/20:5-PC | 0.99731636 | 0.985905 | 0.95527877 | 0.742166 | 0.95784929 | 0.770422 |
| 18:2/18:2-PC | 1.0603074 | 0.659413 | 0.98812756 | 0.939818 | 0.93192555 | 0.661745 |
| 16:0/20:4-PC | 1.06490934 | 0.538807 | 0.99622504 | 0.971327 | 0.93550221 | 0.455385 |
| 18:2/18:1-PC | 0.96840653 | 0.577557 | 0.89092715 | 0.086117 | 0.91999291 | 0.184658 |
| 18:1/18:2-PC | 1.07078251 | 0.398295 | 1.05813699 | 0.488955 | 0.98819039 | 0.885434 |
| 18:2/18:0-PC | 1.06633707 | 0.408941 | 1.06350282 | 0.445368 | 0.99734207 | 0.974566 |
| 18:0/18:2-PC | 1.11833621 | 0.315277 | 0.99485622 | 0.962765 | 0.88958598 | 0.295709 |
| 18:0/18:1-PC | 1.06202441 | 0.718104 | 0.96075544 | 0.805881 | 0.90464535 | 0.482917 |
| 18:1/18:0-PC | 0.88175489 | 0.338807 | 0.88008559 | 0.417603 | 0.99810684 | 0.990254 |
| 18:0/18:0-PC | 1.08363563 | 0.627893 | 0.97409891 | 0.871267 | 0.89891738 | 0.455575 |
| 16:0/22:6-PC | 0.94529988 | 0.649431 | 0.82883788 | 0.150863 | 0.87679888 | 0.269557 |
| 18:2/20:3-PC | 0.96648795 | 0.788704 | 0.86173884 | 0.263761 | 0.89161882 | 0.350593 |
| 18:1/20:4-PC | 1.02533259 | 0.702835 | 0.99577996 | 0.950579 | 0.97117751 | 0.621915 |
| 18:0/20:4-PC | 0.95209803 | 0.499612 | 0.84105477 | 0.026817* | 0.88336992 | 0.112545 |
| 20:3/18:0-PC | 0.84851357 | 0.329646 | 0.78100256 | 0.051967 | 0.92043614 | 0.589735 |
| 18:0/20:3-PC | 1.0244412 | 0.865498 | 0.97825454 | 0.86568 | 0.95491526 | 0.703902 |
| 20:5/20:3-PC | 1.02818733 | 0.826401 | 0.79490689 | 0.144541 | 0.77311485 | 0.104513 |
| 18:1/22:6-PC | 1.04792043 | 0.737998 | 0.84188264 | 0.181728 | 0.80338413 | 0.061796 |
| 18:0/22:6-PC | 0.93695517 | 0.596598 | 0.77512592 | 0.023544* | 0.82728176 | 0.060011 |
| 18:3/16:0-PG | 1.0354231 | 0.751461 | 0.93457106 | 0.483161 | 0.90259823 | 0.351084 |
| 16:1/18:1-PG | 1.08240821 | 0.488128 | 0.93368971 | 0.530843 | 0.86260405 | 0.205514 |
| 16:0/20:5-PG | 0.8834597 | 0.385965 | 0.78877061 | 0.062219 | 0.89282014 | 0.384425 |
| 18:2/18:2-PG | 1.29659149 | 0.069899 | 1.11855258 | 0.408424 | 0.86268697 | 0.228401 |
| 18:1/18:2-PG | 1.13421267 | 0.224511 | 0.96171545 | 0.710502 | 0.84791458 | 0.138972 |
| 16:0/18:0-PS | 0.61426811 | 0.015977* | 0.34491021 | 9.47E-07*** | 0.56149782 | 0.00198** |
| 18:2/18:2-PS | 0.98541374 | 0.916798 | 0.83105455 | 0.217363 | 0.84335596 | 0.260622 |
| 18:0/18:0-PS | 0.89490427 | 0.631139 | 0.47022476 | 0.001306** | 0.525447 | 0.002828** |

The criteria of differential metabolomics based on fold change >2.0 and *p* value <0.05; unpaired student (2-tailed) t tests were used for comparisons between 2 groups, **p*<0.05, ***p*<0.01, ****p*<0.001.

**Supplemental Table S5** Statistical analysis of fatty acid in discovery sets

| **Metabolites** | **CAE versus Controls** | | **CAE versus CAD** | | | **Controls versus CAD** | |
| --- | --- | --- | --- | --- | --- | --- | --- |
|  | **Foldchange** | **P value** | **Foldchange** | | **P value** | **Foldchange** | **P value** |
| 16:0 | 1.00099556 | 0.988135 | | 0.92152438 | 0.275485 | 1.08623883 | 0.230076 |
| 16:1 | 0.99958516 | 0.9975 | | 1.0526853 | 0.693415 | 0.94955744 | 0.702053 |
| 18:0 | 1.01269202 | 0.85119 | | 0.97096971 | 0.661818 | 1.04296973 | 0.500773 |
| 18:1 | 1.07335014 | 0.382499 | | 0.98541605 | 0.862285 | 1.0892355 | 0.305386 |
| 18:2 | 0.93175546 | 0.396025 | | 0.87666585 | 0.085424 | 1.06283991 | 0.447942 |
| 18:3n3 | 1.30186604 | 0.135848 | | 1.19683697 | 0.320933 | 1.08775554 | 0.532941 |
| 20:3n6 | 0.81362837 | 0.046989* | | 0.71896692 | 0.010233* | 1.13166316 | 0.244859 |
| 20:4n6 | 0.75515198 | 0.001265** | | 0.75179295 | 0.001851** | 1.00446803 | 0.949511 |
| 20:5n3 | 0.81770219 | 0.357967 | | 0.61754305 | 0.088944 | 1.32412175 | 0.226238 |
| 22:6n3 | 0.6420695 | 2.43E-05*** | | 0.66427165 | 0.007667** | 0.96657671 | 0.790369 |

The criteria of differential metabolomics based on fold change >2.0 and *p* value <0.05; unpaired student (2-tailed) t tests were used for comparisons between 2 groups, **p*<0.05, ***p*<0.01, ****p*<0.001.

**Supplemental Table S6** VIP value of 35 metabolites of arachidonic acid(AA), eicosapentaenoic acid(EPA) and docosahexaenoic acid(DHA) from PCA analysis in discovery sets

| CAE versus Controls | | | | | | CAE versus CAD | | | | | |
| --- | --- | --- | --- | --- | --- | --- | --- | --- | --- | --- | --- |
| Metabolites | Comp. 1 | Comp. 2 | Comp. 3 | Comp. 4 | Comp. 5 | Metabolites | Comp. 1 | Comp. 2 | Comp. 3 | Comp. 4 | Comp. 5 |
| 4-HDoHE | 0.22951 | 0.22878 | 0.23432 | 0.31683 | 0.36793 | 13-HODE | 1.4949 | 1.4875 | 1.488 | 1.4865 | 1.485 |
| 5-HEPE | 0.54849 | 0.5593 | 0.57461 | 0.57238 | 0.58533 | 15-keto-PGA1 | 1.4424 | 1.4394 | 1.4371 | 1.4364 | 1.4359 |
| 5-HETE | 1.3695 | 1.3471 | 1.3287 | 1.3236 | 1.317 | 12,13-EPOME | 1.4325 | 1.4282 | 1.4282 | 1.4268 | 1.4255 |
| 5s,6r-DiHETE | 0.46406 | 0.45775 | 0.45579 | 0.4707 | 0.51097 | DPA | 1.4012 | 1.3929 | 1.3915 | 1.3905 | 1.3894 |
| 6-trans LTE4 | 0.66148 | 0.65762 | 0.66119 | 0.6597 | 0.6716 | DHA | 1.3821 | 1.3736 | 1.3722 | 1.3713 | 1.3708 |
| 8-HDoHE | 0.80997 | 0.84672 | 0.83612 | 0.83178 | 0.82778 | 5-HETE | 1.3668 | 1.3618 | 1.3609 | 1.361 | 1.3597 |
| 8-HEPE | 0.94362 | 0.95311 | 0.96605 | 0.96181 | 0.96117 | 9,10-EPOME | 1.365 | 1.3621 | 1.3606 | 1.3598 | 1.3587 |
| 9,10-EPOME | 1.3484 | 1.3319 | 1.3216 | 1.3145 | 1.3083 | 15-dexy-12,14-PGD2 | 1.3473 | 1.3426 | 1.3403 | 1.339 | 1.3381 |
| 9-HODE | 0.16715 | 0.33349 | 0.33589 | 0.34117 | 0.38911 | AA | 1.2982 | 1.2908 | 1.2888 | 1.2877 | 1.2864 |
| 9-OXOODE | 0.36985 | 0.46401 | 0.58029 | 0.62945 | 0.62787 | EPA | 1.2758 | 1.269 | 1.2669 | 1.2657 | 1.2646 |
| 10-HDoHE | 0.4521 | 0.48149 | 0.48077 | 0.47771 | 0.48578 | 15-HEPE | 1.2259 | 1.2188 | 1.2177 | 1.2166 | 1.2161 |
| 11,12-EEQ | 0.98419 | 0.96772 | 1.0637 | 1.1031 | 1.0985 | PGD2 | 1.2249 | 1.218 | 1.2157 | 1.2165 | 1.2153 |
| 11-HEPE | 0.71326 | 0.81169 | 0.80824 | 0.80406 | 0.80111 | PGE2 | 1.1981 | 1.1914 | 1.1895 | 1.1904 | 1.1895 |
| 11-HETE | 0.8962 | 0.88711 | 0.87698 | 0.8749 | 0.87648 | 17-HDOHE | 1.1023 | 1.1028 | 1.1018 | 1.1015 | 1.1005 |
| 12,13-EPOME | 1.4217 | 1.3982 | 1.3841 | 1.3741 | 1.3682 | 12-HETE | 1.0154 | 1.0127 | 1.0128 | 1.0137 | 1.0127 |
| 12-HEPE | 0.79783 | 0.85822 | 0.85361 | 0.84764 | 0.8585 | 11,12-EEQ | 0.96962 | 0.97034 | 0.97718 | 0.97713 | 0.97637 |
| 12-HETE | 1.0282 | 1.0161 | 1.0039 | 1.0058 | 1.0009 | 8-HEPE | 0.96007 | 0.96358 | 0.96381 | 0.96348 | 0.96263 |
| 12-keto-LTB4 | 0.97446 | 0.96507 | 1.0099 | 1.0138 | 1.0088 | 12-keto-LTB4 | 0.95626 | 0.95053 | 0.95044 | 0.95071 | 0.95115 |
| 13-HODE | 1.479 | 1.4547 | 1.4392 | 1.4286 | 1.4214 | 15-HETE | 0.90359 | 0.90128 | 0.90046 | 0.90118 | 0.9006 |
| 13OXOODE | 0.95674 | 0.94848 | 0.94755 | 0.94186 | 0.95539 | 11-HETE | 0.88292 | 0.8807 | 0.87929 | 0.88091 | 0.88057 |
| 15-dexy-12,14-PGD2 | 1.3201 | 1.3013 | 1.285 | 1.2773 | 1.2733 | 12-HEPE | 0.82877 | 0.84644 | 0.84846 | 0.84772 | 0.84736 |
| 15-HEPE | 1.2649 | 1.251 | 1.233 | 1.2326 | 1.2265 | 13OXOODE | 0.79951 | 0.80953 | 0.8088 | 0.8087 | 0.82084 |
| 15-HETE | 0.90678 | 0.8924 | 0.88083 | 0.87444 | 0.88093 | 8-HDoHE | 0.77016 | 0.76979 | 0.76862 | 0.76789 | 0.76725 |
| 15-keto-PGA1 | 1.4356 | 1.4114 | 1.3942 | 1.3839 | 1.3769 | 11-HEPE | 0.75116 | 0.78412 | 0.78422 | 0.78461 | 0.7839 |
| 17-HDOHE | 1.1288 | 1.1289 | 1.1171 | 1.1087 | 1.1047 | 18-HEPE | 0.692 | 0.73953 | 0.7431 | 0.74238 | 0.7416 |
| 18-HEPE | 0.66553 | 0.75557 | 0.77031 | 0.76547 | 0.77812 | 6-trans LTE4 | 0.67578 | 0.67172 | 0.67086 | 0.67329 | 0.67325 |
| 19,20-EDP | 0.065842 | 0.10702 | 0.3385 | 0.34897 | 0.35341 | lipoxin A4 | 0.58697 | 0.61852 | 0.61793 | 0.62491 | 0.62687 |
| 20-HDOHE | 0.64279 | 0.6598 | 0.65041 | 0.64613 | 0.65139 | 5-HEPE | 0.5476 | 0.56124 | 0.56127 | 0.56096 | 0.5604 |
| AA | 1.2488 | 1.2278 | 1.2136 | 1.2061 | 1.2023 | 5s,6r-DiHETE | 0.46679 | 0.46709 | 0.4663 | 0.46658 | 0.47837 |
| DHA | 1.3612 | 1.34 | 1.3297 | 1.3197 | 1.3131 | 20-HDOHE | 0.45699 | 0.45639 | 0.45572 | 0.4575 | 0.4571 |
| DPA | 1.329 | 1.3084 | 1.2959 | 1.2864 | 1.2819 | 10-HDoHE | 0.42205 | 0.41958 | 0.41905 | 0.41886 | 0.42043 |
| EPA | 1.2411 | 1.2238 | 1.2087 | 1.2009 | 1.1956 | 9-OXOODE | 0.32315 | 0.33162 | 0.38406 | 0.39395 | 0.3939 |
| PGD2 | 1.1801 | 1.1811 | 1.1695 | 1.1735 | 1.1705 | 4-HDoHE | 0.23395 | 0.24487 | 0.24471 | 0.24603 | 0.24785 |
| PGE2 | 1.2222 | 1.2073 | 1.1939 | 1.1853 | 1.1839 | 9-HODE | 0.088663 | 0.10886 | 0.10869 | 0.10937 | 0.15185 |
| lipoxin A4 | 0.60863 | 0.68022 | 0.71071 | 0.78355 | 0.77958 | 19,20-EDP | 0.085926 | 0.093644 | 0.10377 | 0.11157 | 0.11684 |

**Supplemental Table S7** Change trend of biomarker candidates in validation sets (n=180)

| CAE versus Controls | | | CAE versus CAD | | |
| --- | --- | --- | --- | --- | --- |
| Metabolites | Foldchange | P value | Metabolites | Foldchange | P value |
| PGD2 | 0.639604 | 3.79E-08 | PGD2 | 0.404618 | 7.47E-12 |
| PGE2 | 0.657371 | 2.12E-08 | PGE2 | 0.416719 | 3.9E-12 |
| 15-dexy-12,14-PGD2 | 1.047366 | 0.466089 | 15-dexy-12,14-PGD2 | 0.850914 | 0.172896 |
| 5-HETE | 1.25276 | 0.000127 | 5-HETE | 1.232091 | 0.07 |
| 12-HETE | 1.619251 | 5.14E-07 | 12-HETE | 4.084395 | 3.22E-07 |
| 17-HDoHE | 1.823049 | 9.12E-08 | 17-HDoHE | 7.552359 | 2.64E-07 |
| EPA | 1.433624 | 2.58E-06 | EPA | 2.760066 | 6.72E-07 |
| AA | 1.362783 | 2.6E-07 | AA | 2.179787 | 1.81E-07 |

**REFERENCES**

Blanchard, J. (1981). Evaluation of the relative efficacy of various techniques for deproteinizing plasma samples prior to high-performance liquid chromatographic analysis. *J Chromatogr* 226(2)**,** 455-460. doi: 10.1016/s0378-4347(00)86080-6.

Masoodi, M., Eiden, M., Koulman, A., Spaner, D., and Volmer, D.A. (2010). Comprehensive lipidomics analysis of bioactive lipids in complex regulatory networks. *Anal Chem* 82(19)**,** 8176-8185. doi: 10.1021/ac1015563.

Strassburg, K., Huijbrechts, A.M., Kortekaas, K.A., Lindeman, J.H., Pedersen, T.L., Dane, A., et al. (2012). Quantitative profiling of oxylipins through comprehensive LC-MS/MS analysis: application in cardiac surgery. *Anal Bioanal Chem* 404(5)**,** 1413-1426. doi: 10.1007/s00216-012-6226-x.

Wang, Y., Armando, A.M., Quehenberger, O., Yan, C., and Dennis, E.A. (2014). Comprehensive ultra-performance liquid chromatographic separation and mass spectrometric analysis of eicosanoid metabolites in human samples. *J Chromatogr A* 1359**,** 60-69. doi: 10.1016/j.chroma.2014.07.006.
